# Identification and Expression Profiles of Xyloglucan Endotransglycosylase/Hydrolase Family in Response to Drought Stress in *Larix kaempferi*

**DOI:** 10.3390/plants14121882

**Published:** 2025-06-19

**Authors:** Yan Jiang, Ruodong Qin, Yuqian Wang, Cuishuang Liu, Ying Gai

**Affiliations:** 1 State Key Laboratory of Tree Genetics and Breeding, College of Biological Sciences and Technology, Beijing Forestry University, Beijing 100083, China; jiangyan0430@163.com (Y.J.); 2311210014@bjmu.edu.cn (R.Q.); wangyuqian0089@bjfu.edu.cn (Y.W.); 15615644230@163.com (C.L.); 2 The Tree and Ornamental Plant Breeding and Biotechnology Laboratory of National Forestry and Grassland Administration, Beijing 100083, China

**Keywords:** xyloglucan endotransglucosylase/hydrolase (XTH), *Larix kaempferi*, drought stress, expression analysis

## Abstract

Xyloglucan endotransglucosylase/hydrolase (XTH) is a crucial enzyme in plant cell wall remodeling, which contributes to plant growth, development, and stress response. Based on the transcriptome data of *Larix kaempferi*, this study identified and analyzed 16 *XTH* genes. Sequence alignment and phylogenetic analysis indicated that the *LkXTH* gene family can be divided into three subfamilies, namely the Early Diverging Group, Group I/II, and Group III, all of which share highly conserved motifs and structural features. Expression profiling demonstrated that *LkXTH* genes are actively expressed in the roots, stems, and leaves of *L. kaempferi*. Under drought stress, the expression of *LkXTH1*, *LkXTH2*, *LkXTH3*, *LkXTH4*, *LkXTH6*, *LkXTH14*, *LkXTH15*, *LkXTH17*, and *LkXTH18* increased rapidly in roots. Meanwhile, the expression levels of *LkXTH5*, *LkXTH7*, *LkXTH8*, and *LkXTH13* exhibited significant upregulation in leaves. Notably, *LkXTH11* and *LkXTH16* significantly increased in both roots and leaves, with a more pronounced increase in leaves, but *LkXTH10* displayed significant upregulation in the stems. Furthermore, the heterologous expression of *LkXTH1* and *LkXTH17* in yeast significantly enhances drought tolerance. These findings indicate that individual *LkXTH* genes exhibit distinct organ-specific responses to drought stress, thereby advancing our understanding of their functional roles in larch drought response.

## 1. Introduction

Cell wall reconstruction is a fundamental process in plant growth and development. Xyloglucan, a type of hemicellulose in the cell walls of plants, serves as a key component in cell wall remodeling through fracture and regeneration [1,2,3]. Xyloglucan endotransglucosylase/hydrolase (XTH) is a crucial enzyme in this remodeling process, which plays significant roles in both plant growth and responses to biotic and abiotic stresses [4,5]. By catalyzing the breaking and reconnection of xyloglucan molecules, XTH modifies the cellulose–xyloglucan composite structure of the plant cell wall, allowing for cell wall reconstruction [6,7].

XTH is a large family of enzymes, with its catalytic mechanism divided into two types. The first type, xyloglucan endotransglucosylase (XET) activity, involves XTH binding to the substrate and hydrolyzing it to form a glycosyl–enzyme covalent intermediate. Subsequently, XTH converts the intermediate glycosyl to the reducing end of the polysaccharide residues to carry out the transglycosylation reaction. The second type, xyloglucan endohydrolase (XEH) activity, involves XTH transferring the intermediate glycosyl to a water molecule for hydrolysis [8,9]. The enzymatic kinetics and receptor substrate specificity of XTH have been extensively studied [10,11]. Phylogenetically, XTH family members are classified into three groups, namely Group I/II, Group III, and the Early Diverging Group [12]. A previous study indicated minimal distinction between Group I and Group II, resulting in the formation of Group I/II [13]. Group III can be further divided into two subgroups, IIIA and IIIB [14]. Studies have shown that XTH with glycosyltransferase activity mainly belongs to Group I/II, while XTH with hydrolase activity mainly belongs to Group III. The conserved protein sequence of the XTH family is DEIDFEFLG, which contains amino acid residues with catalytic activity [15,16]. Recent studies have also identified the N101 residue as critical for XET activity [17]. *XTH* genes are widely distributed in various plants species, with members identified in *Arabidopsis thaliana* (33 genes), *Oryza sativa* (29 genes), *Physcomitrella patens* (32 genes), *Sorghum bicolor* (35 genes), *Hordeum vulgare* (24 genes), *Ananas comosus* (24 genes), *Brassica rapa* (53 genes), *Brassica oleracea* (38 genes), *Beta vulgaris* (30 genes), and *Populus simonii* (43 genes) [12,13,14,18,19,20,21,22,23]. However, studies of XTH genes in gymnosperms remain limited. To date, only the enzymatic activities of PrXTH1 in *Pinus radiata* and LkXTH1 in *Larix kaempferi* have been experimentally validated, both demonstrating XET activity [17,24].

Studies of *XTHs* have revealed their spatiotemporal expression specificity, wherein different *XTH* gene family members are expressed in various organizational structures of the *Arabidopsis* root and play diverse roles [25]. Similar observations have been reported in *Schima superba* [26], *Brassica rapa*, and *Brassica oleracea* [12]. Moreover, *XTH* gene expression is affected by environmental stress. Stimuli like touch can increase the expression of *AtXTH22* [27], while the expression of *AtXTH31* is highly responsive to aluminum ion stress treatment. *LsXTH43* regulates the germination process of lettuce seeds, and its expression is significantly upregulated by two times under high temperature stress [28]. Additionally, studies have demonstrated the involvement of *SeXTH* genes in *Salicornia europaea* during its adaptation to abiotic stress reactions [29]. The overexpression of *CaXTH3* in *Arabidopsis* and tomato plants has been found to increase their drought resistance and salt tolerance. Similarly, the overexpression of *PeXTH* in transgenic tobacco stimulates fleshy leaf growth and improves salt tolerance [30,31,32].

Larch is a dominant species in coniferous forests in China. However, little is known about *XTHs* in larch due to the large genome size of gymnosperms. Given the incomplete genome of *Larix kaempferi*, we identified 16 *XTH* genes through transcriptome data analysis. We conducted a comprehensive analysis, including phylogenetic classification, gene structure, conserved motifs, and *cis*-acting regulatory elements. Given previous evidence of XTH involvement in responding to drought stress in other species [30,31], we also examined the expression patterns of these 16 *LkXTHs* genes under drought stress in roots, stems, and leaves. Furthermore, two *LkXTHs* genes containing conserved enzymatic active structures were selected for functional analysis through heterologous expression in yeast. This study provides a theoretical basis for exploring the biological functions of the *LkXTH* gene family and their roles in drought stress response in larch.

## 2. Results

### 2.1. Identification and Characterization of XTHs in L. kaempferi

In this study, we performed a transcriptome-based screening of *XTH* homologs using *A. thaliana XTHs*, *P. patens XTHs*, and *P. tomentosa XTHs* as queries against the *L. kaempferi* transcriptome. We identified a total of 16 *XTH* genes after removing redundant sequences (Table 1). The key characteristics of these genes are presented in Table 1. The length of the LkXTHs ranged from 130 (LkXTH10) to 351 (LkXTH13) amino acids. The theoretical isoelectric point (pI) values for LkXTHs ranged from 4.47 to 9.52 due to differences in the polarity of the constituent amino acids. Moreover, all LkXTHs exhibited negative GRAVY values, indicating hydrophilicity.

The subcellular localization prediction results indicated that all LkXTH proteins were predominantly localized on the cell wall, consistent with their known function. Additionally, five LkXTH proteins were predicted to localize in the cytoplasm, although experimental validation is necessary to confirm this finding.

### 2.2. Phylogenetic Analysis of XTH Proteins

**Figure 1:**
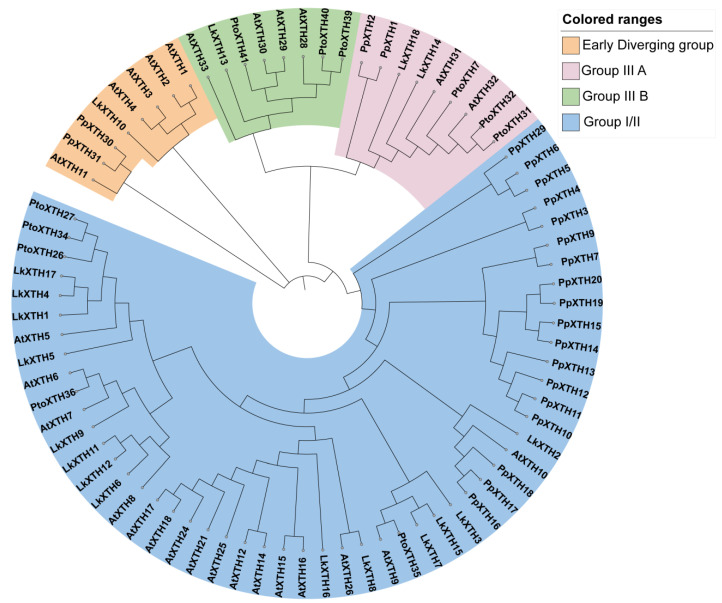
Phylogenetic analysis of XTH family. Species names are abbreviated as follows: Lk, *Larix kaempferi*; At, *Arabidopsis thaliana*; Pp, *Physcomitrella patens*; Pto, *Populus tomentosa*. The phylogenetic tree was constructed using MEGA 11.0 with the maximum likelihood method. The sequences of all XTH proteins are shown in File S1.

To elucidate the evolutionary relationships among XTH gene family members, we constructed a phylogenetic tree (Figure 1). Based on the evolutionary relationships of XTH family members in *A. thaliana*, *P. patens*, and *P. tomentosa*, three groups were identified, namely the Early Diverging Group, Group I/II, and Group III. The Early Diverging Group, comprising eight XTH members, represents the smallest group and is closest to the root. Among the LkXTH family members, only LkXTH10 is in the Early Diverging Group, while LkXTH8 and LkXTH14 belong to Group III A and LkXTH13 belongs to Group III B. The rest of the LkXTH family members (LkXTH1, LkXTH2, LkXTH3, LkXTH4, LkXTH5, LkXTH6, LkXTH7, LkXTH11, LkXTH15, LkXTH16, LkXTH17, and LkXTH18) are classified as Group I/II.

### 2.3. Gene Structures and Conserved Motif Analyses of LkXTH

**Figure 2:**
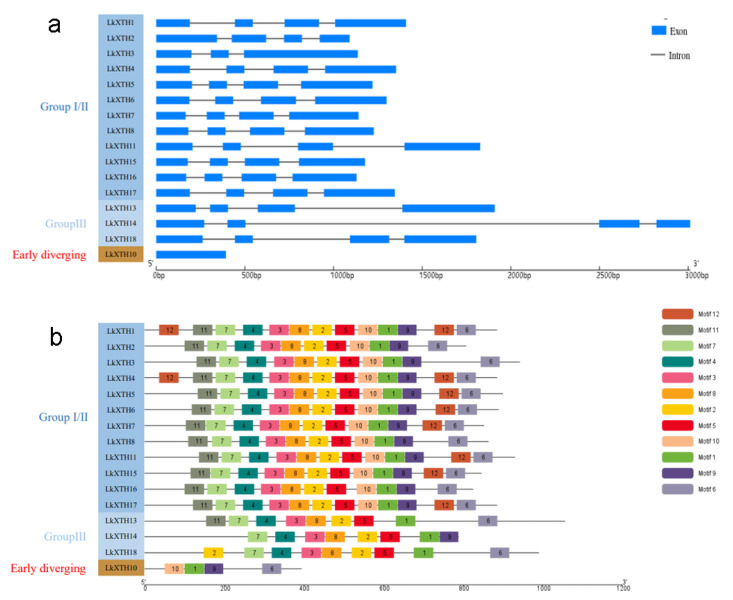
Exon–intron structures (**a**) and protein motif analyses (**b**) of LkXTH proteins.

An exon–intron gene structure diagram (Figure 2a) was constructed based on the incomplete genome sequences (PRJNA588100). Fourteen members of *LkXTH* contain three introns, while *LkXTH3* has two introns, and these insertion sites were also conserved. Notably, *LkXTH10* from the Early Diverging Group completely lacks introns, unlike most other *LkXTH* genes (e.g., *LkXTH3*, *LkXTH5*, and *LkXTH11* in Group I/II, which contain 2–3 introns).

To further investigate the features of *LkXTH* family members, we identified a total of 12 relatively conserved protein motifs within LkXTH proteins (Figure 2b). Motif 1 was present in all genes, while motifs 2, 3, 4, 5, 7, and 8 were observed across all members of Groups I/II and III. Remarkably, motif 4 contains a highly conserved catalytic site DEIDFEFLG, which catalyzes the enzymatic reaction of XET and serves as a characteristic motif of the *XTH* gene family.

### 2.4. Cis-Element Analyses of the Promoter Regions of LkXTH Genes

**Figure 3:**
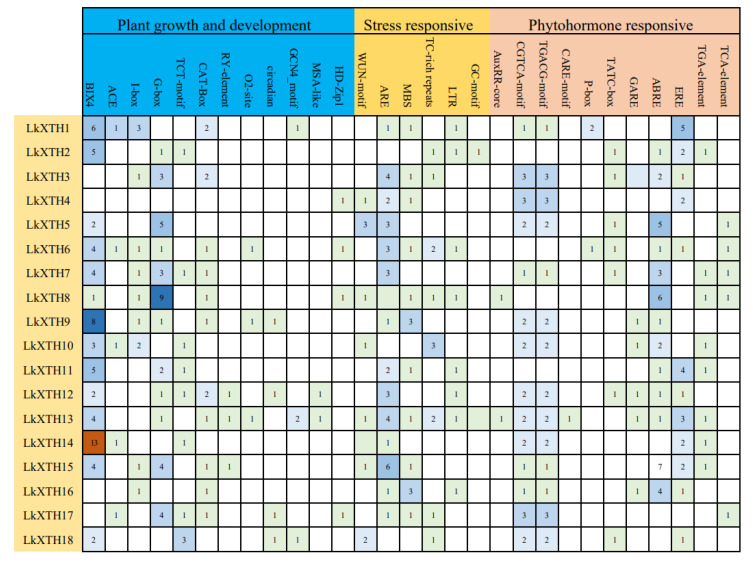
The cis-elements of 16 *LkXTH* genes. Numbers in the table represent the copy numbers of each *cis*-element in individual gene promoters. Color intensity corresponds to the abundance of elements (darker colors = higher counts). Note: This figure is presented in a tabular format for clarity.

Based on the PlantCARE database, *cis*-acting elements of *LkXTH* genes were classified into three groups according to their roles in plant growth and development, stress response, and phytohormone response (Figure 3). Elements associated with plant growth and development include light response elements such as Box 4, ACE, I-box, G-box, and the TCT motif. The meristem expression elements were also identified, including CAT-box, the seed-specific regulation element, the zein metabolism regulatory element (O2 site), the endosperm expression element (GCN4_Motif), the palisade mesophyll cell-related element (HD Zip 1), and the cell cycle regulatory element (MSA-like). Stress response elements included the wound response element WUN-motif, anaerobic induction elements (ARE and GC motif), the low-temperature response element (LTR), the MYB binding site (MBS) involved in drought induction, and the defense and stress response element (TC rich repetition). Additionally, phytohormone-responsive *cis*-elements were detected for auxin (AuxRR and TGA-element), gibberellin (p-box, TATC box, and GARE), methyl jasmonate (CGTCA-motif and TGACG-motif), ethylene (ERE), salicylic acid (TCA-element), and abscisic acid response elements (ABER). All *LkXTH* gene promoter sequences contained members of the three *cis*-element categories. MBS, which is an MYB binding site involved in drought induction, was found in the promoters of 10 *LkXTH* genes, suggesting that this gene family might respond to drought stress.

### 2.5. Expression Pattern Analyses of LkXTH Genes in Different Organs

**Figure 4:**
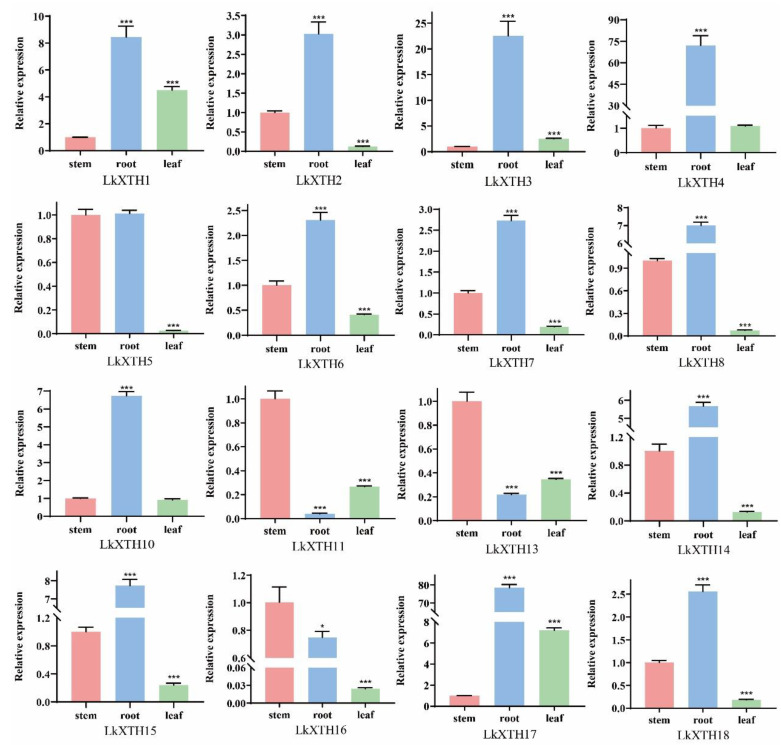
Expression analyses of 16 *LkXTH* genes in different organs including roots, stems, and leaves. The numerical value represents the mean ± SD of three independent replicates. The significant difference was determined through *t*-tests, compared with stems, with * *p* < 0.05 and *** *p* < 0.001.

To investigate the expression pattern of *LkXTHs* in *L. kaempferi*, qRT-PCR was used to analyze the expression level of 16 *LkXTHs* in different organs including the root, stem, and leaf (Figure 4). The results showed that 16 *LkXTH* genes were expressed in different organs, of which 13 genes were highly expressed in roots, but their expression levels were different. The expression levels of *LkXTH2*, *LkXTH6*, *LkXTH7*, and *LkXTH18* were 2.3–3 times higher in roots than stems. The expression levels of *LkXTH1*, *LkXTH8*, *LkXTH10*, *LkXTH14*, and *LkXTH15* were 6–9 times higher in roots than in stems. Compared with the stems, *LkXTH3*, *LkXTH4*, and *LkXTH17* were upregulated 23 times, 72 times, and 78 times in roots, respectively. It is worth noting that the expression level of *LkXTH5* was similar in stems and roots, while the expression level in the leaves was significantly reduced, which was 0.03 times that of the root. Unlike the above, *LkXTH11*, *LkXTH13*, and *LkXTH16* were highly expressed in stems, suggesting a potential significance in regulating stem growth and development.

### 2.6. LkXTH Genes Respond to Drought Stress in Different Organs

**Figure 5:**
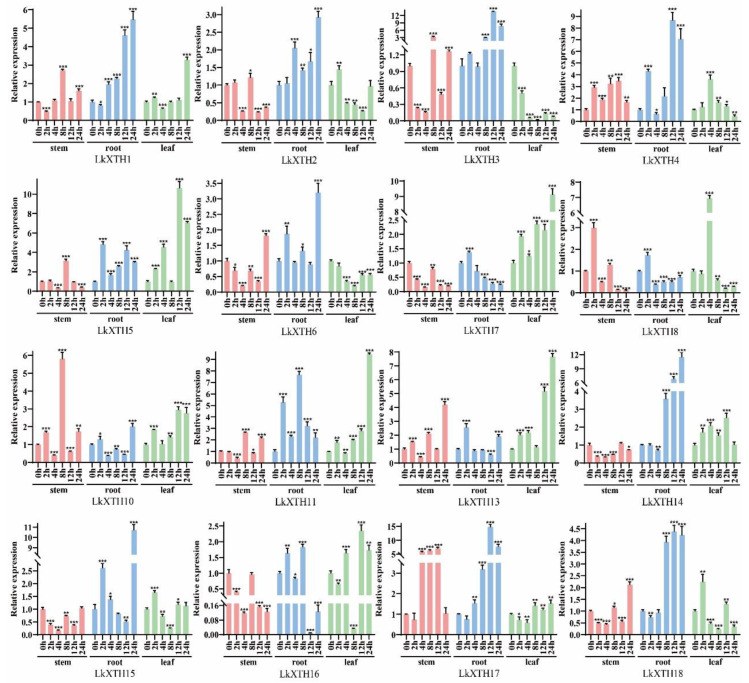
Expression profiles of 16 *LkXTH* genes under drought stress. The numerical value represents the mean ± SD of three independent replicates. The significant difference was determined through *t*-tests, compared with 0 h, with * *p* < 0.05, ** *p* < 0.01, and *** *p* < 0.001.

To investigate the expression patterns of *LkXTH* genes under drought stress, we used qRT-PCR to analyze their expression under drought stress (Figure 5). The results showed that all *LkXTH* genes were expressed differently in organs, with the most significant upregulation observed in roots. The expression of *LkXTH1*, *LkXTH2*, *LkXTH3*, *LkXTH4*, *LkXTH6*, *LkXTH14*, *LkXTH15*, *LkXTH17*, and *LkXTH18* exhibited rapid upregulation in roots over the treatment period, suggesting their active involvement in root responses to drought stress. In contrast, *LkXTH5*, *LkXTH7*, *LkXTH8*, and *LkXTH13* exhibited a significant upregulation in leaves. Notably, *LkXTH5* and *LkXTH7* displayed delayed expression peaks at 24 h, which may be associated with stomatal regulation pathways. Interestingly, a transient downregulation at 8 h was observed for *LkXTH5*, *LkXTH7*, and *LkXTH13* in leaves compared to their 4 h expression levels (Figure 5). This fluctuation may correlate with the photoperiod conditions of the experiment (16 h light/8 h dark), as the 8 h time point coincided with the transition to darkness, potentially influencing circadian rhythm-related gene expression. Additionally, *LkXTH11* and *LkXTH16* were significantly upregulated in both roots and leaves, with a more pronounced increase in leaves. In stems, a temporary decrease in expression was observed at 4 h for *LkXTH10* and *LkXTH16* (Figure 5), possibly reflecting an early stress-responsive adjustment prior to upregulation at later time points. Moreover, *LkXTH10* displayed a significant upregulation in the stem, suggesting that individual *LkXTHs* may play distinct roles in the response to drought stress in different organs.

The investigation further revealed distinct response patterns among *LkXTH* family members under drought stress. In roots, *LkXTH3*, *LkXTH4*, *LkXTH17*, and *LkXTH18* demonstrated significant upregulation after 12 h of treatment, with *LkXTH3* and *LkXTH17* showing 5- and 4-fold increases, respectively. Moreover, the relative expression of *LkXTH1*, *LkXTH2*, *LkXTH6*, *LkXTH14*, and *LkXTH15* reached peak expression at 24 h, while *LkXTH11* and *LkXTH16* displayed their highest relative expression levels at 8 h. These findings suggest that members of the *XTH* gene family respond differently to drought stress and play distinct roles in different tissues.

### 2.7. Osmotolerance of Yeast Transformants

**Figure 6:**
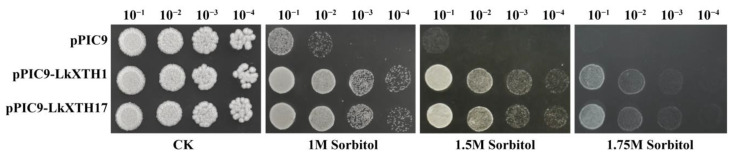
Growth status of yeast with the empty vector (pPIC9) or the recombinant vector (pPIC9-LkXTH1/17) under osmotic stress. The control (CK) represents yeast cells grown on normal YPD medium. 1 M Sorbitol: solid YPD plates supplemented with 1 M sorbitol; 1.5 M Sorbitol: solid YPD plates supplemented with 1.5 M sorbitol; 1.75 M Sorbitol: solid YPD plates supplemented with 1.75 M sorbitol. The results shown are reproducible across three independent experiments. Serial 10-fold dilutions of yeast cultures (e.g., 10^−3^ = 1000× dilution) were spotted on YPD plates with 0, 1, 1.5, or 1.75 M sorbitol and incubated at 28 °C for 48 h. Data represent three independent biological replicates.

To evaluate the drought resistance conferred by *LkXTH* genes, *LkXTH1* and *LkXTH17* were selected for heterologous expression in yeast due to their pronounced drought induction under drought stress, phylogenetic proximity to XET-active orthologs, and the presence of the conserved N101 catalytic residue. On standard YPD medium, yeast strains expressing *LkXTH1* or *LkXTH17* exhibited similar growth to those carrying the empty vector. However, under osmotic stress conditions induced by 1.0 M, 1.5 M, and 1.75 M sorbitol, the recombinant yeast strains demonstrated markedly improved growth compared to the control (Figure 6). These results suggest that *LkXTH1* and *LkXTH17* enhance osmotic tolerance in yeast, thereby improving their drought stress resistance.

## 3. Discussion

XTH, as a key enzyme in plant cell wall remodeling, plays an important physiological role in plant growth, development, and response to environmental stress [16]. We identified a total of 16 *XTH* genes in *L. kaempferi* based on transcriptome data, which were divided into three groups, namely the Early Diverging Group, Group I/II, and Group III. The Early Diverging Group contains one *LkXTH*, while Group III contains three *LkXTHs*. The number of genes in Group I/II is higher than that in both Group III and the Early Diverging Group, with a total of 12 *LkXTHs*. These results are consistent with findings from other plant species including *H. vulgare* and *A. comosus* [13,20]. LkXTH1, LkXTH4, and LkXTH17 from Group I/II were found to be on the same branch as PtoXTH27 and PtoXTH34, which were previously confirmed to have XET activity [33]; therefore, these three LkXTH members probably exhibit XET activity. Other members of the *XTH* gene family are likely inactive in terms of catalytic activity.

XTH proteins possess a highly conserved catalytic domain, characterized by the motif (H/W/R)-(D/N)-E-(I/L/F/V)-D-(F/I/L/M)-E-(F/L)-(L/M)-G [15,16], which was identified in all LkXTHs. However, only LkXTH1, LkXTH4, and LkXTH17 contain the conserved N101 site, a key residue implicated in substrate binding and XET activity [17]. This finding aligns with our phylogenetic analysis, further supporting that these three LkXTH proteins as the most likely candidates within the larch XTH family to exhibit XET activity. Thus, LkXTH1 and LkXTH17 were selected for functional validation based on their predicted enzymatic activity, with expression patterns under drought stress providing additional evidence for their involvement in stress adaptation. While catalytic activity (XET/XEH) is a hallmark of XTH function, non-catalytic members may serve structural or regulatory roles. For instance, AtXTH22 lacks enzymatic activity but contributes to cell wall remodeling through protein–protein interactions [27]. The absence of catalytic motifs in some LkXTHs (e.g., LkXTH10) suggests potential roles in signaling or scaffold formation, warranting further investigation.

All LkXTH proteins were predicted to localize to the cell wall, which is consistent with their known function. Interestingly, five XTH proteins from Group I/II were also predicted to localize to the cytoplasm (Table 1). This dual localization pattern has been reported in other species. For instance, in *A. thaliana*, 23 AtXTH proteins are predicted to localize to the cell wall, while 10 are predicted to localize to both the cell wall and cytoplasm [14]. Similarly, in *P. trichocarpa*, 20 PtXTHs were predicted to localize to the cell wall and 18 XTH proteins in both compartments [26]. On the other hand, the Plant-mPLoc server gives only the prediction of the LkXTH proteins. Therefore, the cytoplasm localization for some LkXTH proteins could be due to the absence of a signal peptide, as previously reported in the case of XTH29 of Arabidopsis thaliana [34]. AtXTH29 was located in the plant cell, but it reached its final destination of the cell wall with an unconventional protein secretion (UPS) [34]. Similarly to AtXTH29, the cytoplasm-localized LkXTHs may ultimately function in the cell wall despite lacking classical signal peptides.

Gene structure analysis revealed conserved insertion sites among *LkXTHs*, consistent with findings in *Arabidopsis* [14,16]. Unlike Group I/II members (e.g., *LkXTH3*, *LkXTH5*, *LkXTH11*) with 2–3 introns, *LkXTH10* in the Early Diverging Group is intronless. This intronless structure is rare in angiosperms but has been reported in *P. patens* [23].

*Cis*-acting regulatory elements act as molecular switches in the transcription regulation of gene expression. *Cis*-element analyses suggest the potential involvement of *XTH* family genes in abiotic stress and hormone response. The stress response elements related to low temperature, light, injury, and drought were identified in all members of the *LkXTH* gene family, underscoring the impact of external stressors on the expression of *LkXTH* genes.

The *XTHs* exhibit tissue- and organ-specific expression patterns [26]. In this study, 16 *LkXTH* genes were expressed in the roots, stems, and leaves of larch. Among them, 13 *LkXTH* genes were highly expressed in the roots, while three *LkXTH* genes were highly expressed in the stems. After exposure to drought stress, nine *XTH* genes exhibited rapid upregulation in the roots, whereas four genes exhibited significant upregulation in the leaves. Notably, two *LkXTH* genes were induced significantly in both the roots and leaves. The transient downregulation of leaf-expressed *XTH* genes at 8 h (Figure 5) may be linked to photoperiodic regulation. Larch seedlings were grown under a 16 h light/8 h dark cycle, and the 8 h time point of drought treatment coincided with the onset of darkness. Previous studies have shown that *XTH* gene expression can be modulated by circadian rhythms in *Arabidopsis* [25], suggesting that light/dark cycles might interact with drought stress responses in larch. Moreover, *LkXTH10* showed significant upregulation in the stem, indicating diverse functional roles. The dip in stem gene expression at 4 h (Figure 5) could represent a brief phase of metabolic adjustment before the activation of stress-responsive pathways. Similar transient expression patterns have been reported in *Populus* under osmotic stress, where early energy reallocation precedes the induction of protective genes [32]. Notably, a similar organ-specific expression pattern exists in *XTH* gene families of other species like *S. europaea* L. [29]. The XTH activity in the root meristem tissue region of *Durum Wheat* seedlings was significantly increased following drought treatment [35]. Similarly, in the study of XTH function, the upregulation of *GmXTH1* in soybean seedlings has the potential to enhance water absorption by increasing the total root length, surface area, total projection area, root volume, average diameter, total cross number, and total root tip number [36]. In addition, the overexpression of *CaXTH3* would affect stomatal movement and lead to stomatal closure, which might reduce transpiration water loss under dehydration stress [31], and *PagXTH12* in poplar was upregulated under drought, improving drought resistance in overexpression lines [37]. The overexpression of *PeXTH* in tobacco affects the cell wall remodeling in defense-related cells, reduces leaf water loss and stomatal aperture, and improves plant drought resistance [32], paralleling the functional role of *LkXTH1* and *LkXTH17* in yeast osmotic tolerance. Although yeast cell walls lack xyloglucan (composed mainly of mannans and β-1,3-glucans), the observed increase in osmotolerance following the heterologous expression of *LkXTH1* and *LkXTH17* may result from indirect mechanisms. Jiang et al. demonstrated that yeast expressing XET-active *PtoXTH27/34* exhibited increased intracellular total sugar content and altered polysaccharide molecular weight profiles, suggesting a potential modulation of sugar metabolism or broader stress adaptation pathways [33]. While XTHs primarily act on xyloglucan in plants, their heterologous expression in yeast may trigger conserved stress-responsive mechanisms such as osmotic adjustment. Nonetheless, the precise biochemical changes induced by *LkXTH1* and *LkXTH17* in yeast require further investigation. These findings suggest that the XTH genes play a significant role in enhancing plant drought resistance through the catalytic modification of cell wall polysaccharides, as well as through potential involvement in signaling pathways or structural remodeling.

## 4. Materials and Methods

### 4.1. Plant Material and Treatments

*L. kaempferi* seeds from Liaoning province were germinated in a greenhouse at 23 °C with a 16/8 h (day/night) photoperiod. The seedlings of *L. kaempferi* grew well for 70 days (5–6 cm high) and then were transferred in liquid MS medium for 48 h. After that, the seedlings were treated with liquid MS medium (control group) and liquid MS medium added with 20% PEG 6000 (experimental group, 20% PEG 6000 simulates drought stress) for 0, 2, 4, 8, 12, and 24 h, respectively. Three larch seedlings were combined as one biological replicate group. Three groups were taken at each period of each treatment, and their roots, stems, and leaves were harvested. The collected experimental materials were immediately frozen in liquid nitrogen and then stored at −80 °C for further analyses.

### 4.2. Identification of XTH Genes in L. kaempferi

XTH sequences of *A. thaliana* and *P. patens* were obtained from the phytozome (Phytozome V13 Doe.gov) website. The genome data PRJNA588100 [38] and transcriptome PRJNA648500 [39] used in this study were obtained from the NCBI database. Gene identification was based on transcriptomic data (PRJNA648500) due to the absence of a complete *L. kaempferi* genome. Using 33 *A. thaliana* XTH protein sequences and 31 *P. patens* XTH protein sequences as templates [14,23], two methods were used to identify the members of the XTH family in *L. kaempferi*. First, BLASTp with an expected value (E-value) less than 1 × 10^5^ was used to search for potential LkXTHs. Second, the hidden Markov model (HMM) of the XET C-terminus domain (PF06955) and the glycosyl hydrolase family-16 domain (PF00722) were obtained from the Pfam database (http://pfam.xfam.org/, accessed on 7 January 2022). Then, the Simple HMM Search tool from TBtools software (version 1.108) was used to search for potential LkXTHs [40]. Phmmer Search (https://www.ebi.ac.uk/Tools/hmmer/search/phmmer, accessed on 7 January 2022) and CD Search (https://www.ncbi.nlm.nih.gov/Structure/cdd/wrpsb.cgi, accessed on 7 January 2022) were used to analyze the conserved domains of the candidate protein sequences and identify whether they have the conserved domains of the XTH protein. The Plant-mPLoc server (http://www.csbio.sjtu.edu.cn/bioinf/plant-multi/, accessed on 14 January 2022) was used to predict the subcellular location of LkXTH proteins [41]. ProtParam (http://web.expasy.org/protparam/, accessed on 14 January 2022) was used to analyze the physical and chemical parameters of the LkXTH proteins [42].

### 4.3. Phylogenetic Analysis

The XTH protein sequences of *A. thaliana* [23], *P. patens* [14,15], and *P. tomentosa* [33] were downloaded from the Phytozome13 (https://phytozome.jgi.doe.gov/pz/portal.html, accessed on 18 March 2022) and NCBI (https://www.ncbi.nlm.nih.gov, accessed on 18 March 2022) databases (File S1). The sequence alignment of XTH proteins was performed using ClustalW (version 2.1) [43]. We then used MEGA 11 software (version 11.0.13) to construct a phylogenetic tree based on the maximum likelihood method.

### 4.4. Conserved Motif and Gene Structure Analyses

MEME (version 5.5.1) [44] was used with default parameters and was visualized using TBtools (version 1.120) [40] to predict and analyze the conserved motifs of the LkXTH proteins (sequences provided in Supplementary File S1). Exons and introns of each *XTH* were obtained by locating the CDS sequence of *LkXTH* in the genomic information of *L. kaempferi* [38] in NCBI and visualized using GSDS 2.0 (https://gsds.gao-lab.org, accessed on 7th April 2023) [45].

### 4.5. Cis-Regulatory Element Analyses

The 2000 bp upstream sequences of the starting codon of 16 *LkXTH* genes were obtained from the *L. kaempferi* genome [38]. *Cis*-regulatory elements were identified and analyzed using PlantCARE (http://bioinformatics.psb.ugent.be/webtools/plantcare/html/, accessed on 7 April 2023) with default parameters [46].

### 4.6. RNA Extraction and Quantitative Real-Time PCR Expression Analyses

The total RNA of the roots, stems, and leaves of *L. kaempferi* were extracted by the Plant RN38 Kit (Aidlab, Beijing, China) and digested with DNase I at 42 °C for 15 min to remove genomic DNA by TRUEscript RT MasterMix (OneStep gDNA Removal) Kit (Aidlab, Beijing, China). RNA integrity and concentration were verified by agarose gel electrophoresis and NanoDrop 2000. Then, cDNA was synthesized with Reverse-transcription Kit (Aidlab, Beijing, China), according to the manufacturer’s instructions. The specific primers used were designed by Primer Premier 5.0 software (Table S1).

qRT-PCR was performed using a 7500 Fast Real-Time PCR system (Applied Biosystems, Foster, CA, USA) with SYBR Premix Ex TaqTM (Aidlab, Beijing, China). qRT-PCR was performed in a 25 μL reaction volume containing 1 μL cDNA (equivalent to 50 ng total RNA), 12.5 μL 2× SYBR Green qPCR Mix (Aidlab, Beijing, China), 1 μL of each primer (10 μM), and 9.5 μL RNase-free H_2_O. The reaction program was set as follows: 95 °C for 30 s, followed by 40 cycles at 95 °C for 5 s and 60 °C for 30 s. The *α-tubulin* gene was selected as the internal reference after preliminary validation showed stable expression across tissues and drought treatments, consistent with its use in conifer stress studies [47]. Melting curve analysis (60–95 °C) confirmed primer specificity, with single peaks observed for all amplifications. Three independent biological replicates were performed and each biological replicate contained 3 technical replicates. Finally, the relative expression level of genes was calculated using the 2^−ΔΔCT^ method. All statistical analyses were conducted by *t*-tests in GraphPad Prism 8 software. Data normality was assessed using the Shapiro–Wilk test, and significant differences between the control and treatment groups at each time point were determined by two-tailed Student’s *t*-tests (*p* < 0.05). No multiple testing correction was applied due to independent pairwise comparisons.

### 4.7. Osmotolerance of Yeast Transformants

Yeast (*Pichia pastoris*) was selected as a heterologous expression system due to its rapid growth, genetic simplicity, and established utility for plant gene functional analysis [33,47]. Recombinant plasmids pPIC9-*LkXTH1* and pPIC9-*LkXTH17* were constructed and separately transformed into *P. pastoris* GS115, with the empty pPIC9 vector serving as the control. Single colonies from each transformant were selected and cultured for 48 h under induction conditions. The OD_600_ value of each induced culture was measured, and the cell suspension was adjusted to OD_600_ = 1.0 [33].

To assess osmotolerance under drought-mimicking conditions, serial 10-fold dilutions of each yeast culture were prepared using sterile water. A 4 μL aliquot from each dilution was spotted onto YPD agar plates supplemented with 0 M (control group), 1.0 M, 1.5 M, or 1.75 M sorbitol. Each treatment was performed in triplicate. Plates were incubated at 28 °C for 48 h, after which colony growth was observed and compared across all treatment groups.

## 5. Conclusions

In this study, 16 *XTH* genes were identified in *L. kaempferi* and classified into three groups, namely the Early Diverging Group, Group I/II, and Group III. The number of genes in Group I/II was significantly higher than those in Group III and the Early Diverging Group. Furthermore, all LkXTHs were predicted to localize to the cell wall, consistent with their known function. Expression profiling revealed that all 16 *LkXTH* genes were expressed in the roots, stems, and leaves. After drought stress, *LkXTH1*, *LkXTH2*, *LkXTH3*, *LkXTH4*, *LkXTH6*, *LkXTH14*, *LkXTH15*, *LkXTH17*, and *LkXTH18* were rapidly upregulated in roots, while *LkXTH5*, *LkXTH7*, *LkXTH8*, and *LkXTH13* were significantly induced in leaves. Notably, *LkXTH11* and *LkXTH16* showed strong induction in both roots and leaves, particularly in leaves. In contrast, *LkXTH10* exhibited a marked upregulation in stems. This organ-specific expression pattern under drought stress suggests functional diversification among *LkXTH* family members. Moreover, the heterologous expression of *LkXTH1* and *LkXTH17* in yeast significantly enhanced drought tolerance, highlighting their potential roles in abiotic stress resistance. These findings provide a foundation for the further functional characterization of *XTH* genes in larch and offer candidate genes for improving drought tolerance in forest trees.

## Figures and Tables

**Table 1:** Molecular characteristics of 16 LkXTH proteins from *L. kaempferi*.

Symbol	Genes ID	Peptide Length (aa)	Molecular Weight (kDa)	Isoelectric Point (pI)	GRAVY	Subcellular Localization
LkXTH1	OP235906	294	33.8114	8.71	−0.470	Cell wall and cytoplasm
LkXTH2	OP235907	268	30.2061	5.72	−0.199	Cell wall
LkXTH3	OP235908	313	35.4489	8.58	−0.342	Cell wall
LkXTH4	OQ746059	294	33.7183	8.88	−0.451	Cell wall and cytoplasm
LkXTH5	OP235909	299	34.7603	8.50	−0.520	Cell wall and cytoplasm
LkXTH6	OP235910	295	34.2441	4.79	−0.395	Cell wall
LkXTH7	OP235911	283	32.4874	5.51	−0.280	Cell wall
LkXTH8	OQ746060	287	32.7222	4.47	−0.362	Cell wall
LkXTH10	OQ746061	130	14.6555	8.96	−0.624	Cell wall
LkXTH11	OQ746062	309	35.5432	8.15	−0.449	Cell wall
LkXTH13	OQ746063	351	39.3741	6.07	−0.405	Cell wall
LkXTH14	OQ746064	262	30.2513	9.52	−0.388	Cell wall
LkXTH15	OQ746065	281	31.6536	4.91	−0.219	Cell wall
LkXTH16	OP235912	274	31.0488	5.00	−0.332	Cell wall and cytoplasm
LkXTH17	OP235913	294	33.6462	8.21	−0.416	Cell wall and cytoplasm
LkXTH18	OP235914	329	37.3069	7.65	−0.316	Cell wall

## Data Availability

All data reported in this study can be found in the manuscript file. Publicly available genome data PRJNA588100 and transcriptome data PRJNA648500 can be found in the NCBI database.

## Supplementary Materials

The following supporting information can be downloaded at: https://www.mdpi.com/article/10.3390/plants14121882/s1; Supplementary Material File S1: Information of all XTH protein sequences that were used to construct the phylogenetic tree. Supplementary Material Table S1: qRT-PCR primers.
