# Supplementary material for: Identification and Expression Profiles of Xyloglucan Endotransglycosylase/Hydrolase Family in Response to Drought Stress in Larix kaempferi
Source: Plants (Basel). 2025 Jun 19;14(12):1882. doi: 10.3390/plants14121882 (PMC12196611; doi:10.3390/plants14121882)
Supplement: Supplementary file 1 [file plants-14-01882-s001.zip › Supplementary material Table S1.pdf]

Article title: Identification and Expression Profiles of Xyloglucan Endo-transglycosylase/Hydrolase Family in Response to Drought Stress in *Larix kaempferi*
Journal name: Plant Cell Reports
Author names: Yan Jiang, Ruodong Qin, Yuqian Wang, Cuishuang Liu and Ying Gai
Affiliation of the corresponding author (Ying Gai): State Key Laboratory of Tree Genetics and Breeding, College of Biological Sciences and Technology, Beijing Forestry University, Beijing 100083, P. R. China; The Tree and Ornamental Plant Breeding and Biotechnology Laboratory of National Forestry and Grassland Administration, National Engineering Laboratory for Tree Breeding, Beijing, 100083, P. R. China.
E-mail address of the corresponding author (Ying Gai): gaiying@bjfu.edu.cn

Supplementary material Table S1. qRT-PCR primers

Gene	Forward primer (5'→3')	Reverse primer (5'→3')
Tubulin	ATTATGAAGAGGTCGGAGCAG	CCCCACCAGTACTACCTATCAA
LkXTH1	ATTCAACCAACCCATGAAAATAT	CCACCATCGACGGCCTA
LkXTH2	TGTCCAACTCTTGGTCGTCG	TCCCTTTGTCAGCGCAGTAG
LkXTH3	ACAAGCAGCAGAGTGAAGAAT	GAGGATCGGTCAAGTAGGAGT
LkXTH4	CTTCCAGTCCAAGGGTAGC	CAGCGTTCTGGGAGGAT
LkXTH5	TCATTCTCCTACTCCATTCTGCTG	AAGTGCCCAAATAAATATGTCCC
LkXTH6	AGAGGAGGGCTGGACAA	GGCAGTAATCATAGACGAGGA
LkXTH7	GGTGGGATATGCCCTCTTATT	TCAGGTGGCGAAGTTGGA
LkXTH8	TCTGTGAATGGACTGGC	ACTGTAGTTGTCCTTGCTG
LkXTH10	ATGGTGGCTGGAGTTGTT	CTTGCATAGGCTTCGATG
LkXTH11	AGATTATGTGGGCTCAGGA	AGGCTGTCCGCTTCTATT
LkXTH13	GACGGACCAACGCACAG	TCGATAGCGAGACAACGAA
LkXTH14	GCTTCATACATTACTGCTCCTT	TAATAGCCGCACTGAAGTAG
LkXTH15	GTGCAGCCTGGTAGTGG	TCGCCTTGGGAAGACAT
LkXTH16	GTCACTGCCTATTATCTATCGTCTCA	GCTTGATGTCCAGAGCACGG
LkXTH17	TGGTGGATGAAGTGCCG	AAGCTGCGACGAAGGGA
LkXTH18	CATAAACGGGACAGGAGA	TCAGTAGCCCAAGAGGAG
